# Navigation of a New-Onset Pulmonary Embolism in a Patient Receiving Female-to-Male Gender-Affirming Care: A Case Report

**DOI:** 10.7759/cureus.63101

**Published:** 2024-06-25

**Authors:** Danielle C Thor, Krut Patel, Monisha Singh

**Affiliations:** 1 Internal Medicine, Jefferson Health New Jersey, Stratford, USA

**Keywords:** venous thromboembolism (vte), pulmonary embolism (pe), hematology, gender-affirming care, transgender medicine, lgbtq medicine, lgbtq

## Abstract

The growth of the transgender and/or gender diverse (TGD) community has created an increased demand for comprehensive and evidence-based gender-affirming care, especially when providing a hormone-based regimen. Although there are known risks of adverse events from receiving exogenous hormone replacement therapy, many of these risks are typically attributed to members of the TGD community receiving estrogen-based therapy. In this report, a case of a subsegmental pulmonary embolism in a male patient assigned female at birth who was actively receiving testosterone-based, female-to-male gender-affirming care is detailed. In doing so, attention is drawn to a potential complication of this approach, thereby empowering clinicians and patients alike to be cognizant of such risks while still pursuing this otherwise pivotal and necessary care.

## Introduction

The medical management of members of the Lesbian, Gay, Transgender, Queer, Intersex, Asexual, Two-Spirit, and all others (LGBTQIA2S+) community remains an evergrowing element of Western healthcare. The LGBTQIA2s+ community encompasses approximately 7% of the United States adult population, and within this cohort, approximately 1.6% identify as transgender or gender diverse [1]. The World Professional Association for Transgender Health (WPATH) defines transgender and gender diverse (TGD) individuals as members of the varied communities globally of people with gender identities or expressions that differ from the gender socially attributed to the sex assigned to them at birth [2]. As the TGD community slowly approaches greater societal recognition and acceptance, more of its members are turning to the medical establishment to seek gender-affirming care, or the utilization of medical and surgical interventions to recognize and affirm a person's gender identity [2]. In doing so, a continuous effort must be made by all clinicians who may serve the TGD community to better understand the benefits of this affirmatory care while also navigating any associated risks. The WPATH’s most current version of “Standards of Care for the Health of Transgender and Gender Diverse People, Version 8,” at the time of publication provides a detailed appendix of common adverse events associated with estrogen-based and testosterone-based therapies [2]. These guidelines are considered to be the industry standard in gender-affirming care, but they have yet to identify cases of thromboembolic events in TGD persons receiving testosterone-based therapy. In the case presented below, a female-to-male transgender patient presented with a novel adverse event of subsegmental pulmonary embolism from their testosterone-based gender-affirming care.

## Case presentation

A 21-year-old male assigned female at birth with a past medical history of moderate persistent asthma and bipolar disorder (type II) presented to the emergency department with complaints of shortness of breath. The patient received both primary care and gender-affirming care from a local comprehensive LGBTQIA2S+ treatment center and was maintained on testosterone-based hormone replacement therapy for approximately three months at the time of admission. On examination, the patient described a sudden onset of shortness of breath and chest tightness while at work and originally sought care at an urgent care center for what he believed to be a recurrent asthma attack. However, following evaluation by urgent care personnel, the patient was directed to their local emergency department for further workup of a possible blood clot.

**Figure 1:**
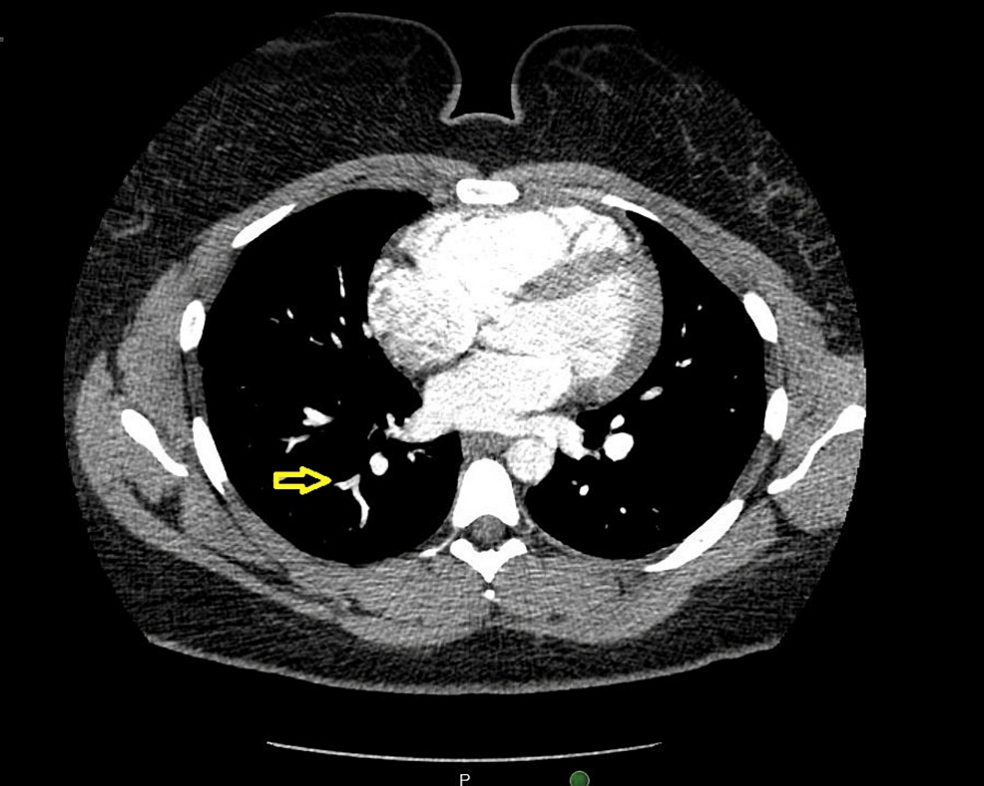
CTA of the chest with a small pulmonary embolism of the right lower lobe branching distal pulmonary artery segment

**Figure 2:**
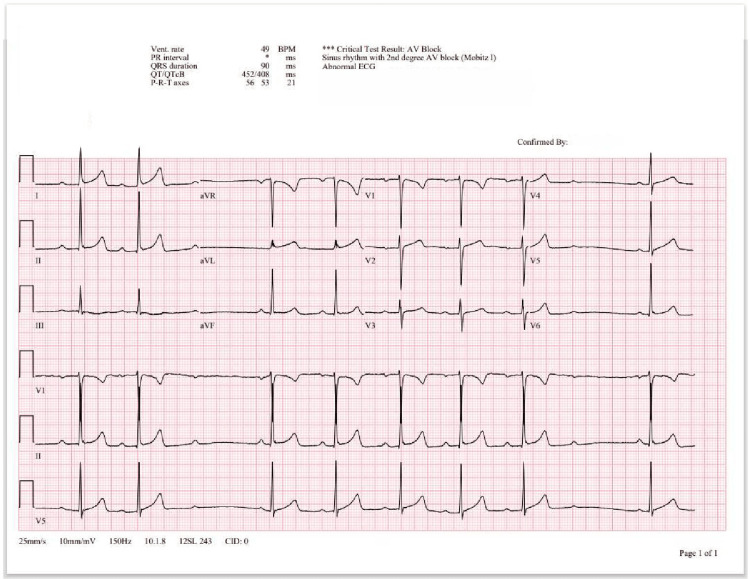
ECG at the time of admission with normal sinus rhythm and second degree AV nodal block (Mobitz type I)

In the emergency department, the patient was found to be febrile with a maximum temperature of 102.3°F, but otherwise hemodynamically stable and not requiring supplemental oxygen. His laboratory workup was notable for creatinine of 1.31 mg/dL (baseline: 0.8-0.9 mg/dL) and D-Dimer of 530 ng/mL (baseline: < 243 ng/mL); his basic metabolic panel and complete blood count (including hemoglobin, hematocrit, and platelet count) were within normal limits. Venous dopplers of the bilateral lower extremities were negative, but a computed tomography angiography (CTA) of the chest was notable for a small pulmonary embolism (PE) of the right lower lobe branching from the distal pulmonary artery segment (Figure 1). In addition, an electrocardiogram (ECG) obtained from the patient at the time of admission was notable for sinus rhythm with second-degree AV nodal block (Mobitz type I) (Figure 2). A transthoracic echocardiogram was complete and was negative for right-heart strain or other acute findings.

The patient was admitted to telemetry and his PE was treated with a continuous heparin infusion before eventual transition to a direct oral anticoagulant (DOAC). Cardiology was consulted for further recommendations on the management of the AV nodal block on admission. The AV block was no longer seen on repeat ECG tracings and therefore warranted conservative measures with outpatient management. Hematology/oncology was consulted for consideration of a hypercoagulability workup given the patient’s young age and noted that the patient’s body mass index (38%) and concurrent use of hormone replacement therapy are possibly causal factors for his PE. The patient was stabilized and discharged with outpatient follow-up with his primary care provider (PCP) and pulmonologist in place. His hormone replacement therapy was held on admission and eventually resumed by his PCP following clearance from pulmonology. Furthermore, his hypercoagulability workup was negative and his primary care provider and pulmonologist ultimately decided to discontinue his DOAC following the completion of a six-month anticoagulation course. 

## Discussion

As the medical community continues to develop its understanding of the care needs of LGBTQIA2S+ individuals, further contextualization of treatment paradigms and risk stratification should follow. When focusing on those who identify as TGD, the increases in visibility and/or social acceptance of TGD persons will undoubtedly result in an increase in the provision of gender-affirming care. Gender-affirming care has already repeatedly demonstrated the ability to mitigate a variety of negative mental health outcomes, including depression, anxiety, and suicidality [3-4]. Prominent medical societies beyond WPATH, such as the American Academy of Family Physicians [5], the American Academy of Pediatrics [6], and the Endocrine Society [7], have all codified guidelines detailing standards of gender-affirming care. Through these advances, the greater argument is made of the benefits of gender-affirming care for TGD individuals as a necessary service to help these patients live their healthiest, fullest lives. However, the practical administration of such care requires a holistic understanding of the risks and benefits of the associated medical interventions.

In the case presented here, a 21-year-old male who was assigned female at birth presented with a new-onset PE with limited risk factors outside of their baseline obesity and use of testosterone-based hormone replacement therapy (HRT). A minimal but real risk of venous thromboembolism (VTE) with estrogen-based HRT is already documented in the literature and associated guidelines for gender-affirming care [2,8-9]. However, limited information outside of the details of this case is available for episodes of VTE in persons receiving testosterone-based HRT. The theoretical risk of aromatization exists when one is exposed to large volumes of testosterone, as the testosterone excess is easily shunted towards the physiological pathway for estradiol and/or estrogen formation [10]. This phenomenon has been documented in cases surrounding cis-gendered male patients seeking HRT for low testosterone [11], but less so in the TGD community. Given this patient’s recent onset of testosterone-based HRT and predisposition for hyperestrogenism with their body habitus, it is likely that their HRT resulted in instances of accidental aromatization, thereby generating the hypercoagulable state necessary for VTE formation. 

Although the patient's gender-affirming care was postponed out of an abundance of caution pending multidisciplinary medical clearance, it is worth noting that this pause may not be necessary for similar scenarios. In this case, the patient’s otherwise minimal clot burden and ability to tolerate both heparinization and subsequent transition to a DOAC may be sufficient treatment for the prevention of future VTE events without withholding gender-affirming care. Continual arguments are made that the benefits of gender-affirming care on reductions in suicidality or self-harm outweigh the risks of VTE or other adverse effects [12-13]. Ultimately, clinicians who interact with members of the TGD community on active HRT should understand and communicate the risks associated with such therapy but otherwise feel empowered to continue gender-affirming care despite any potential non-critical adverse events.

## Conclusions

This case report details a transgender male undergoing testosterone-based gender-affirming care whose experience was complicated by a subsegmental pulmonary embolism. In doing so, a novel complication to testosterone-based HRT is highlighted, but discontinuation of gender-affirming care is discouraged due to its greater positive impact on suicidality and other mental health outcomes in the TGD community. Although the strength of this case report is limited by the fact that it represents one patient, it still serves a greater purpose in furthering the medical understanding of the potential complications surrounding gender-affirming care.
